# Metastasis of bronchogenic carcinoma to the 5^th ^metacarpal bone: a case report

**DOI:** 10.1186/1757-1626-1-284

**Published:** 2008-10-30

**Authors:** Alexandros Tzaveas, Georgios Paraskevas, Ioannis Pazis, Anastasios Dimitriadis, Panagiotis Kitsoulis, Aristeidis Vrettakos

**Affiliations:** 1 Orthopaedic Department, B' IKA-ETAM "Panagia" Hospital, Thessaloniki, Greece

## Abstract

**Background:**

Metastatic lesions to the hand are very rare and represent 0.1% of all osseous metastases.

**Case presentation:**

We present a patient with metastasis of bronchogenic carcinoma of the lung to the 5^th ^metacarpal to draw the attention for the potential of such lesions to be developed in this region. Due to the extensive metastasis to the hand the patient was referred to the oncologists.

**Conclusion:**

The surgeon should be cautious regarding the differential diagnosis, the usual poor prognosis of such patients and the questionable need for reconstructive surgery.

## Background

Metastases to the hand are rare events with around 200 cases reported in the literature [1-6]. They comprise only 0, 1% of all osseous metastases [4]. The terminal phalanges are the most frequent site of metastasis, followed by the metacarpals and the proximal phalanges [3,4]. We report a metastasis of bronchogenic lung cancer to the 5^th ^metacarpal.

## Case presentation

**Figure 1:**
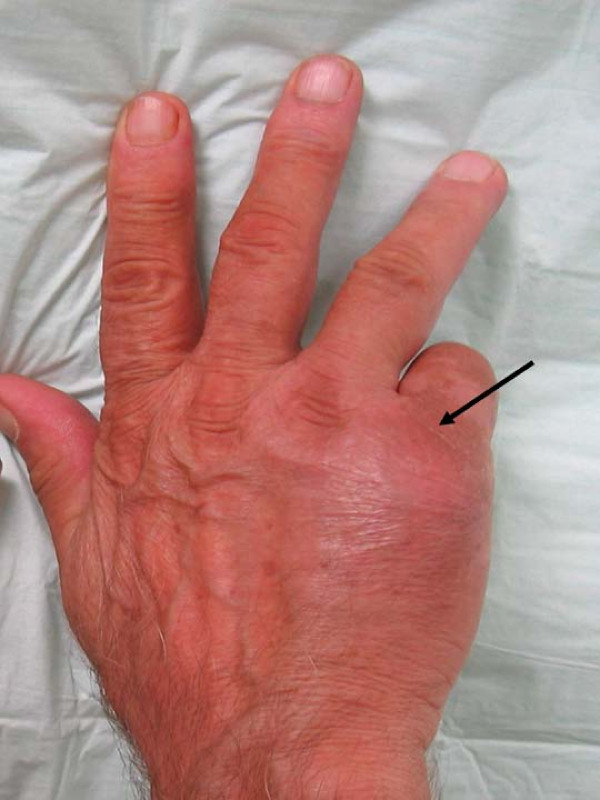
The swelling at the dorsal part of the hand over the 5^th ^metacarpal.

**Figure 2:**
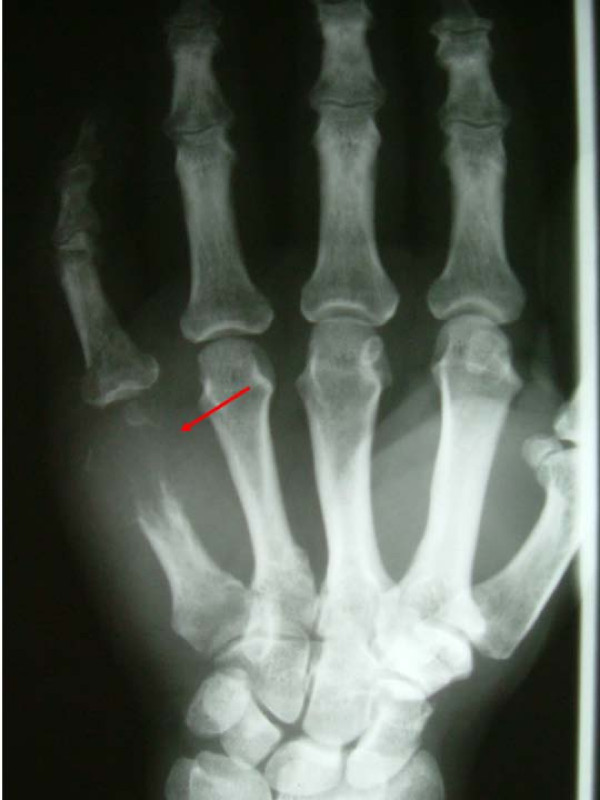
Destruction of the distal part of the 5^th ^metacarpal.

**Figure 3:**
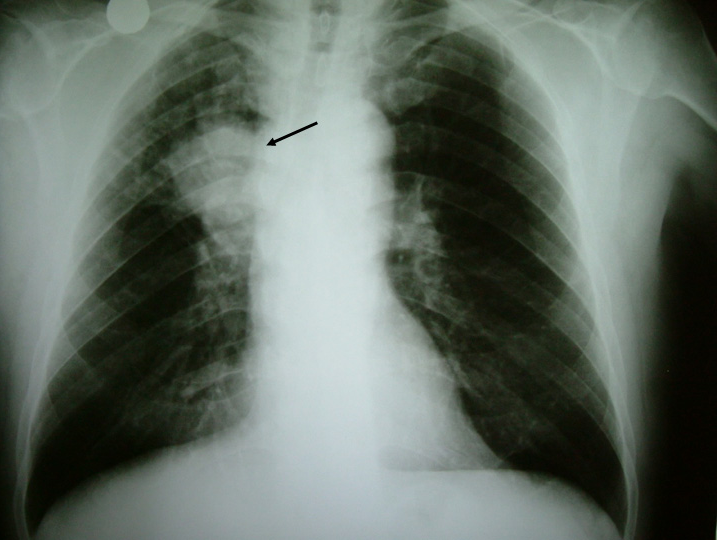
The x-ray finding of the bronchogenic carcinoma.

**Figure 4:**
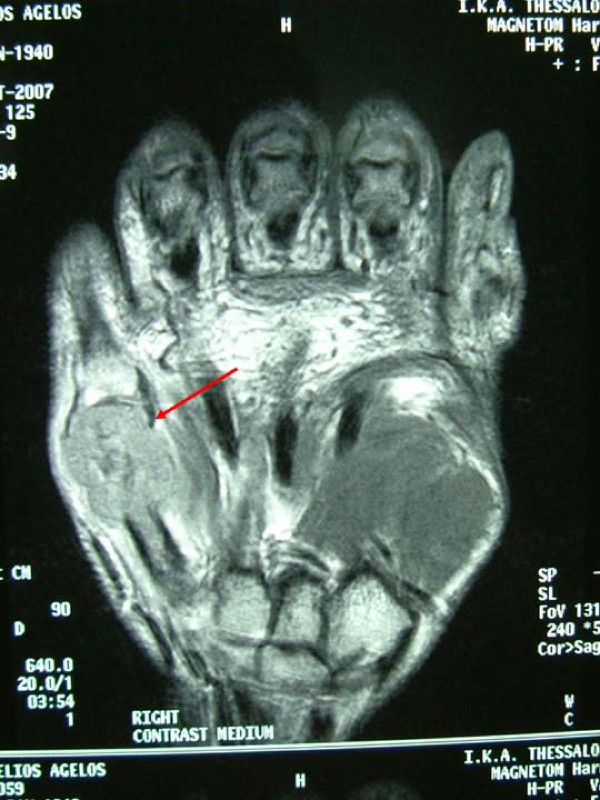
A solid mass at the area of 5^th ^metacarpal.

A 68 year old male patient was presented to the outpatient department with a painful swelling on the dorsum of the right hand without any history of trauma. It was primarily considered to be cellulitis and treated with antibiotics for 10 days by the general practitioner. He had bronchogenic lung carcinoma which was diagnosed 5 months ago. He also had a bone scan done which did not reveal any metastases. Clinical examination revealed a palpable mass at the ulnar part of the dorsum of the hand (Figure 1). The hand was warm, red and tender. Flexion and extension movements of the fingers were painful. There was no neurovascular deficit. No epitrochlear or axillary lymph nodes were palpable. The radiographs revealed a lytic lesion of the fifth metacarpal with destruction of its distal part (Figure 2). Chest x-ray showed a large mass in the right upper lobe of the lung, in keeping with a bronchogenic carcinoma (Figure 3). MRI of the hand revealed a large, solid mass originating from the fifth metacarpal and extending to the adjacent soft tissues (Figure 4). Open biopsy showed metastatic carcinoma. The case was discussed with the Oncologist. We decided that, due to the broad extension of the mass to the hand, the patient should not receive any surgical treatment and he was referred to the oncology department.

The most common site of metastatic deposits to the hand is the distal phalanx. The incidence of metastasis to the metacarpals is 17%, phalanges 66% and carpal bones 17% [7]. Keramidas and Brotherston [8] published a case with metastasis to both metacarpal and carpal bones, which is extremely rare.

It is rare to see bronchogenic carcinoma metastases to the bones distal to the elbow and knee. Floridis was the first to describe this rare entity in the United States in 1934 [9]. Depass (1958) was the first to report a metacarpal metastasis in the English literature [9].

The lung is the most common source with 42% followed by the breast and kidney each of which account for 11% [3,7]. Other sources include colon, prostate, thyroid, oesophageal and bone cancers [10]. Men are more commonly involved than women [8]. Metastases in the hand are the first clinical sign of an undiagnosed tumor in only 16% of all metastases [8].

The exact reason for this rarity of such metastases is not known. In 1889 Paget [9] suggested the "seed and soil" theory for metastasis, which states that one needs to have both seed (i.e. tumor emboli) and good soil (or proper site) for this tumor emboli to settle down and grow. Prostaglandins have been implicated as possible chemotactic factors that influence cell migration and adherence to the skeleton [5]. Tumor deposits occur mainly in the bones hematopoietically active and multiply to produce typical lytic lesions or, occasionally, formation of reactive bone [9]. The infrequent development of metastases to the hand may be related to the smaller amount of red marrow present in these bones. Piney [9] observed, as early as 1922, the absence of bone marrow in phalanges – spared metastasis. Recent trauma and increased blood flow have also been implicated as a nidus for tumor metastasis [1,3,4]. Joll [9] suggested that repeated trauma might play a role in reducing the local tissue resistance thus producing a fertile ground for "seed". Shinz [9] pointed out that primary malignancies erode veins (systemic or portal) and tumor emboli are filtered by lung or liver. But in the case of bronchogenic carcinoma, the tumor erodes the pulmonary vein and thus has access to systemic circulation and, consequently, widespread metastasis. In contrast to this, the vertebral venous plexus of Batson permits mostly axial skeleton metastases [9].

The patient usually presents with a painful, swollen, erethymatous and warm hand [8,9]. The x-rays show lytic bony lesion. The differential diagnosis includes gout, pulp space infection, osteomyelitis, septic arthritis, rheumatoid arthritis, tenosynovitis and reflex sympathic dystrophy [11].

The prognosis of these patients is poor, with the median survival being usually six months [8]. This should be taken in account in the management of these patients. Radiotherapy and chemotherapy may be appropriate and amputation could be an option for lesions at the distal phalanx. Reconstructive surgery is not indicated due to poor prognosis [8].

## Conclusion

The surgeon should have a high level of clinical suspicion when examining patients with the above symptomatology and concomitant malignancies or lytic lesions and age over 45 years [12]. Management of such cases should be multifactorial, due to the rarity and the poor prognosis. The cooperation of the orthopaedic surgeon with the radiologist, the pathologist and the oncologist is imperative for the accurate diagnosis and the avoidance of overtreatment and unnecessary reconstructive surgery.

## Consent

Written informed consent was obtained from the patient for publication of this case report and any accompanying images. A copy of the written consent is available for review by the Editor-in-Chief of this journal.

## Competing interests

The authors declare that they have no competing interests.

## Authors' contributions

AT and IP examined the patient for the first time and on his follow ups. AT and GP were involved in reviewing the literature. AD and AV were involved in the research of the importance of our finding and the interpretation of the finding. AT and GP were responsible for final proof reading of the article. All authors read and approved the final manuscript.
